# Prospecting salivary tau as a diagnostic for Alzheimer’s type dementia

**DOI:** 10.1590/1980-5764-DN-2024-0253

**Published:** 2025-04-28

**Authors:** Gustavo Alves Andrade dos Santos, Francisco de Assis Carvalho do Vale, Valeria Paula Sassoli Fazan

**Affiliations:** 1 Universidade de São Paulo, Faculdade de Medicina de Ribeirão Preto, Departamento de Anatomia e Cirurgia, Ribeirão Preto SP, Brazil.; 2 Faculdade São Leopoldo Mandic de Araras, Faculdade de Medicina, Departamento de Medicina, Araras SP, Brazil.; 3 Universidade Estadual de Campinas, Faculdade de Engenharia de Alimentos, Laboratórios de Nutrição e Metabolismo, Campinas SP, Brazil.; 4 Universidade Federal de São Carlos, Departamento de Medicina, São Carlos SP, Brazil.

**Keywords:** Biomarkers, Saliva, Tau Proteins, Alzheimer Disease, Enzyme-Linked Immunosorbent Assay, Biomarcadores, Saliva, Proteínas Tau, Doença de Alzheimer, Ensaio de Imunoadsorção Enzimática

## Abstract

Although recognized as one of the most critical and prevalent dementias, Alzheimer’s disease (AD) still does not have an affirmative biological marker, being diagnosed by excluding other pathologies that present similar symptoms. In the last decade, advances have been made in researching pathophysiological phenomena related to AD, with the identification of probable biomarkers. Some of these substances have a direct action on the degenerative activity that involves AD and may be associated with other pathological processes, such as inflammation and diabetes.

**Objective:**

These occurrences led us to investigate further whether the levels of certain substances may be associated with the onset and progression of Alzheimer’s disease. Biomarkers can be found in plasma, saliva and cerebrospinal fluid.

**Methods:**

This project investigated tau protein as a possible salivary biomarker in 76 patients, control group and Alzheimer’s, with different age groups, to establish a positive correlation between the studied biomarker and AD.

**Results:**

Our findings showed that phosphorylated tau (pTAU) concentrations are higher in AD patients and somewhat lower in elderly patients without Alzheimer’s, but in young patients without Alzheimer’s the levels are much lower. Total tau had very similar levels in three groups evaluated.

**Conclusions:**

Based on these results, we believe in the possibility of using saliva as an auxiliary method in diagnosing Alzheimer’s disease, with the advantages of low cost, non-invasiveness, and ease of collection. Still, more investigations will be needed to confirm this method presented.

## RESUMO

Embora reconhecida como uma das demências mais críticas e prevalentes, a doença de Alzheimer (DA) ainda não possui um marcador biológico afirmativo, sendo diagnosticada pela exclusão de outras patologias que apresentam sintomas semelhantes. Na última década, avanços foram feitos na pesquisa de fenômenos fisiopatológicos relacionados à DA, com a identificação de prováveis biomarcadores. Algumas dessas substâncias têm ação direta na atividade degenerativa que envolve a DA e podem estar associadas a outros processos patológicos, como inflamação e diabetes.

**Objetivo:**

Essas ocorrências nos levaram a investigar mais a fundo se os níveis de certas substâncias podem estar associados ao início e à progressão da doença de Alzheimer. Os biomarcadores podem ser encontrados no plasma, saliva e líquido cefalorraquidiano.

**Métodos:**

Este projeto investigou a proteína tau como possível biomarcador salivar em 76 pacientes, grupo controle e Alzheimer, com diferentes faixas etárias, para estabelecer uma correlação positiva entre o biomarcador estudado e a DA.

**Resultados:**

Nossos achados mostraram que as concentrações de proteína tau hiperfosforilada (pTAU) são maiores em pacientes com DA e um pouco menores em pacientes idosos sem Alzheimer, mas, em pacientes jovens sem Alzheimer, os níveis são muito menores. O tau total apresentou níveis muito semelhantes nos três grupos avaliados.

**Conclusões:**

Com base nesses resultados, acreditamos na possibilidade de utilizar a saliva como método auxiliar no diagnóstico da doença de Alzheimer, com as vantagens de baixo custo, não invasividade e facilidade de coleta. Mais investigações serão necessárias para confirmar esse método apresentado.

## INTRODUCTION

The number of people with Alzheimer’s disease in the world could reach 74.7 million by 2030 and 131.5 million by 2050, due to the aging population. This scenario shows that the disease characterizes a global health crisis that must be addressed^1-3^.

A biomarker is verified objectively and understood as “a type of parameter of physiological functions considered normal, states that configure disease or consequences of exposure or aggression”^4,5^.

In people with confirmed Alzheimer’s dementia, the β1-42 peptide is reduced in the cerebrospinal fluid, while total tau (t-tau) and phosphorylated tau (pTau) proteins are elevated^6^. Currently, a type of tau protein, recognized as phosphorylated, is the most investigated site in clinical studies seeking to identify a biomarker related to AD^7^.

Recently, saliva has been proposed as a potential source of biomarkers due to its accessible collection and ability to be used in the diagnosis and risk assessment of several pathological conditions^8^.

The feasibility of using saliva to identify biomarkers is known, and it may be predictive for the diagnosis of Alzheimer’s^9^. The concept of Salivomics has been proposed in view of the significant advances in the understanding of saliva, which include genomic, transcriptomic, proteomic, metabolomic aspects and microRNA analysis^10,11^. The salivary proteome contains around 2,300 proteins, of which a third are identical to plasma proteins^12^. Biomarkers have been found in saliva and are related to AD, with varying results^8,11,13,14^.

The current biomarkers investigated with potential for diagnosing Alzheimer’s (Table 1) are obtained through body fluids, which may be easier or harder to obtain, depending on other variables such as cost and practicality in collection^16,33^.

**Table 1:** Fluids and biomarkers in Alzheimer’s disease.

Diagnostic source	Evidence
Cerebrospinal fluid (CSF)^13,15-19^	Presence of Aβ42 (amyloid beta protein fraction 42 amino acids), pTau (phosphorylated tau protein), t-tau (total tau protein)^13,16-19^, p-tau181(phosphor tau-181)^15,19^, p-tau-231^19^.
Imaging Biomarkers^20,21^	Temporal lobe atrophy^22^, contrast between gray matter and white matter^23^, Hippocampal atrophy^24^, presence of neurofibrillary tangles (NFTs)^21^.
Functional Imaging (use of ^18^F-fluorodeoxyglucose-positron emission — FDG-PET)	Reduced glucose uptake in temporoparietal regions in addition to the posterior cingulate cortex^25^; use of tracers such as florbetapir, florbetaben, and flutemetamol show amyloid protein deposition^26,27^.
Blood^18,28,29^	Aβ40, Aβ42, tau proteins^18,28,29^.
Saliva^9,30-32^	Aβ42^9,30-32^, pTau^9,30,31^, t-tau^9,30,31^, lactoferrin^30,31^.

### Saliva

Approximately 99% of human saliva is water, and it is estimated that a healthy person can produce 600 mL of this fluid daily; however, while we sleep, this quantity drops to almost zero^34^. Saliva is a unique colloidal fluid comprising proteins and other large molecules with distinct rheological and lubricating properties. Scientists have permanently studied the origin and composition of human saliva based on experience in biological, physiological, and dental research^35-37^.

Recently, a review panel analyzed the relationship between possible salivary biomarkers and the respective identification techniques, emphasizing the Sandwich enzyme-linked immunosorbent assay (ELISA) and the Luminex ELISA^14^. It was found that the Aβ42 protein, in the Sandwich ELISA method, appears increased in patients with AD versus patients in the control group^37^; in the Luminex ELISA, the Aβ42 protein was not detected in the AD group^38^, but in the Nanobead ELISA group it appeared increased when compared to the control^39^; the Aβ40 protein did not register changes in the Sandwich ELISA and Nanobead ELISA^40^; in the Single Molecule Arrays (SIMOA), Sandwich ELISA and Luminex ELISA methods, t-tau did not register changes in both groups^38^; however, the p181/T-tau ratio appeared to be increased in the AD group^38^; and in the Western blot, an increase in s396/T-tau was found in the AD group^12^.

The first study showed significantly elevated levels of Aβ42 in the saliva of AD patients, as it was able to quantify the salivary and plasma concentrations of Aβ40 and Aβ42 peptides in AD patients and controls^40^. Increased salivary Aβ42 concentrations in AD patients compared to controls have also been observed and confirmed in other studies^41^. Mass spectrometry and Luminex assays, which are highly sensitive procedures, were used to evaluate the salivary level of t-tau, p-tau and amyloid β42 in AD. The conclusion obtained by these studies concluded that the p-tau/t-tau ratio was significantly higher in AD patients when compared to controls^1,38^.

Total tau and pTau181 in saliva are detectable and shown to be altered in patients with AD dementia and mild cognitive impairment (MCI). Low concentrations of t-tau have been observed in patients with AD, and higher concentrations (low or high) of pTau181 have also been observed in patients with MCI and AD dementia^15^.

We sought to confirm salivary tau as an initial killer, and therefore we believe that it may also be a substance present in MCI. It will also be possible to determine whether salivary p-tau/t-tau is abnormally elevated in individuals with frontotemporal dementia (FTD), a type of tauopathy^42^.

The aim of this study was to demonstrate that saliva, through the evaluation of the concentrations of t-tau and pTau proteins, can be quite useful in the diagnosis of Alzheimer’s dementia.

## METHODS

This case-control study was conducted in patients with a probable diagnosis of AD and cognitively healthy patients without AD, in three research centers located in Brazil.

### Legal aspects of the research

The research was submitted to Plataforma Brasil in compliance with the determinations of Resolution 466 of the National Health Council (CNS), of December 12^th^, 2012, protocol number: 057867/2018.

All participants and/or their respective legal guardians received clarification regarding the objectives of the research, and then acceptance was considered after signing the Free and Informed Consent Form (FICF).

### Ethics approval

This study was performed in line with the principles of the Declaration of Helsinki. Approval was granted by the Ethics Committee of Universidade de São Paulo, Faculty of Medicine, Ribeirão Preto, Brazil, in December 24, 2018, number 90628918.1.0000.5440

### Patient groups and exclusion and inclusion criteria

The organization and division of volunteers without AD and patients with AD followed the inclusion and exclusion criteria determined by legal norm 13 of 2017 of the Ministry of Health, which establishes the clinical protocol and therapeutic guidelines for AD in Brazil. All participants had a confirmed diagnosis or were healthy. We selected patients of both sexes, seeking to balance the quantities proportionally, as will be demonstrated below. The same applied to age, when recruiting participants, age ranges were defined, as will be described.

Seventy-six participants took part in this research project, with the following arrangement:

- Group of elderly people without a diagnosis of Alzheimer’s dementia: 26 cognitively healthy elderly individuals, 13 women and 13 men, aged between 65 and 80 years, without a diagnosis of AD;
- Adults without AD: 25 adults, without a diagnosis of AD, aged between 19 and 59 years, randomly selected by the researcher based on clinical characteristics and because they did not have a diagnosis of AD. There were 13 women and 12 men;
- Alzheimer’s disease group: 25 elderly people with probable AD; aged between 65 and 80 years, clinically selected by the team doctor; there were 13 women and 12 men.

Some important considerations regarding the eligibility of volunteer participants:

- Patients, selected in proportional quantities to avoid disproportion between the number of men and women. Before the start of recruitment, we sought to obtain quantities of men and women in similar numbers, after which there was no change in the final quantities;
- No differentiation has been established regarding the stage of the disease in patients diagnosed with probable Alzheimer’s;
- The diagnosis for AD was based on a report issued by the team physician, who considered the following parameters: results of neuropsychological evaluations: Mini-Mental State Examination (MMSE) and Clock Drawing Test, imaging (Magnetic Resonance Imaging), laboratory tests (B12, thyroid function, serological test for neurosyphilis);
- Research participants without signs of mouth injury until salivary fluid collection.

### Sample characterization

All participants, whether diagnosed with Alzheimer’s dementia or healthy, had their selection randomized based on inclusion and exclusion criteria. At the time of collection, all volunteers were numerically coded (01 to 76), and their data was protected and kept confidential. The division of groups regarding gender was performed randomly, following the volume of adhesions to the project.

### Biological and clinical analysis

For safety reasons, we collected all samples in plastic tubes, such as colorless Eppendorf Pressure, and measuring 1.5 mL, which are less susceptible to leaks, facilitating the entire process, from checking handling and pipetting. We added enough dry ice to ensure that the material remained frozen during transport.

### Tau protein

**Figure 1:**
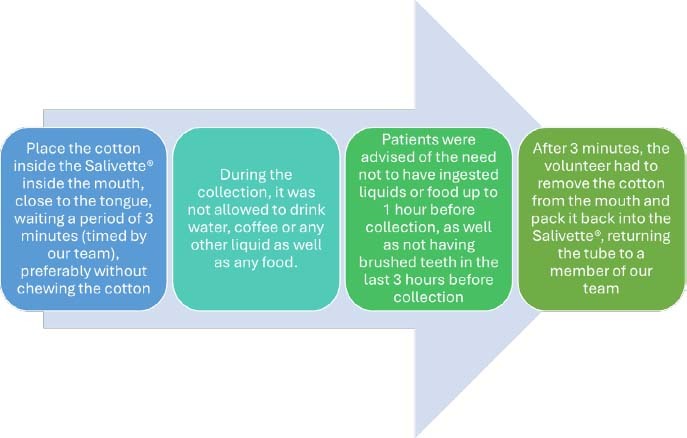
Schematization of the saliva collection process in participants.

Saliva was collected using Salivette® bottles from the company SARSTEDT, consisting of a plastic tube containing a cotton roll. For the collection to be carried out correctly, the following information was provided to the research participants (Figure 1).

**Figure 2:**
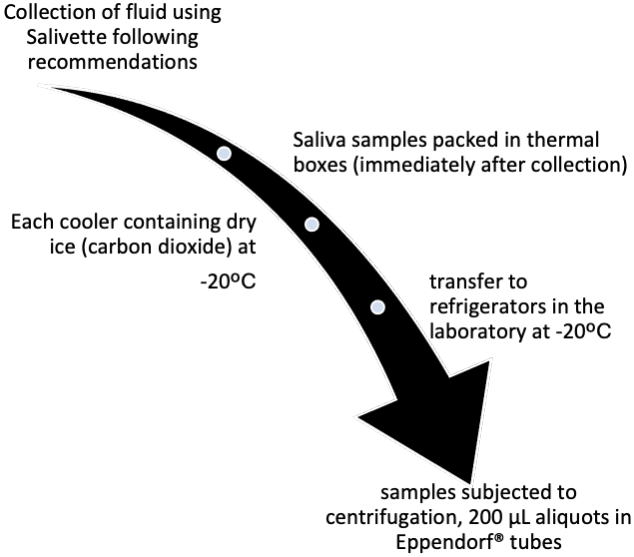
Sample processing.

After being removed from the freezing temperature (freezer), in a laboratory environment, saliva samples were subjected to centrifugation for five minutes at 3 thousand revolutions per minute (rpm). In the sample processing phase, saliva was separated into 200 μL aliquots in Eppendorf® tubes for quantification of tau protein using the ELISA method (Figure 2).

To quantify t-tau and pTau by the ELISA method in saliva, the research participants used, respectively, the MBS022635 Human Tau Protein ELISA Kit and E-EL-H5314 Human pTau. In the statistical analysis, we used the Kruskal-Wallis Test.

## RESULTS

**Figure 3:**
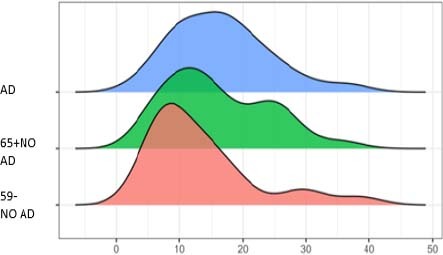
Total tau protein concentrations in saliva.

**Figure 4:**
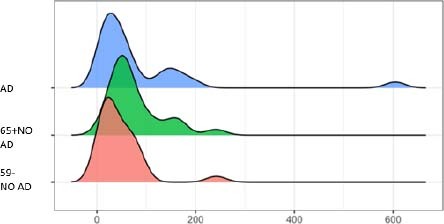
Hyperphosphorylated tau.

Figures 3 and 4 show the concentrations of total tau (T1T) and hyperphosphorylated tau (TP) proteins found in the saliva of patients/research participants. We can observe that the total tau protein levels do not show evidence of differences in the concentrations of the three age groups of patients; that is, in patients with and without AD, total tau protein does not appear to be a probable biomarker to aid in the diagnosis of the disease. However, the pTau protein brings an exciting finding, showing variations between young and elderly patients with and without the disease. Our results show that pTau was elevated in the elderly, and in the elderly with AD it was even higher.

The mean results for total tau protein across groups in Table 2 show little or no variation. However, hyperphosphorylated tau presents high levels in the group of patients with Alzheimer’s disease, a slightly lower level in the elderly without AD, and much lower in the young group without AD. The detection of tau proteins (T and P) was robust and assertive at levels compatible and coherent with the presence or absence of AD.

**Table 2:** Distribution of tau protein concentrations in groups.

	59- No AD		65+ No AD		AD		p-value*
	Mean	SD	Mean	SD	Mean	SD	
tTau	18.23	25.60	16.00	8.04	16.58	7.66	0.257
pTau	47.28	48.75	172.7	493.3	180.4	495.3	0.035

Abbreviation: AD, Alzheimer’s disease; SD, standard deviation; Mean, Average;

Note:*Kruskal-Wallis test.

We performed “two by two” comparisons of all areas, with the average indicated in Table 3. This “average” value is considered poor, although we will see in Graph 3 that only the groups of elderly patients, with and without AD, are below 0.5.

**Table 3:** Receiver Operating Characteristic curve.

	AUC
TP	0.5691

Abbreviation: TP, true positive.

Spikes in the graphs indicate that many people are around that value. In the total tau group (T1T), we noticed that the distribution of the 59- No AD group shifted further to the left than the others, indicating that subjects in this group typically present lower values. Significantly few subjects also exceed 20 units, which is much more common in the other two groups. For the hyperphosphorylated tau (TP) group, the group of patients over 65 years old without AD, it is slightly shifted to the right, indicating that its individuals generally have higher values. As in T1T, the group of healthy participants, under 59 years of age, rarely presents values above 100, which are relatively common in the other groups.

We performed a search for cutoff points to maximize class statistics to obtain sensitivity/specificity generalization for more than two groups (Table 4).

**Table 4:** Cutoff values to split group (levels: 59- No Alzheimer’s disease—AD, 65+ No AD, AD).

	Direction	Cutoffs
TP	increasing	32.895, 127.770

Note: Metric: Youden’s J index.

## DISCUSSION

Post hoc comparisons were made in the analysis of the overall data and are expressed by the results of pairwise group comparison procedures using the Steel-Dwass-Critchlow-Fligner (SDCF) method (Table 5). Since our analysis was on t-tau and p-Tau, this procedure was only relevant for p-Tau. We have no evidence to differentiate t-tau between the groups, so there is no point in making pairwise comparisons. For p-Tau, we have evidence to say that the three groups are not all the same, and by making a pairwise comparison, we have evidence that the two control groups are different from each other.

**Table 5:** Steel-Dwass-Critchlow-Fligner pairwise comparison of ranks for phosphorylated tau.

	t	p-value
59- No AD x 65+ No AD	3.457	0.039
59- No AD x AD	0.820	0.831
65+ No AD x AD	2.657	0.145

An important issue concerns the power of the test; however, calculating it after the sample has already been collected based on the test results gives the same information as the p-value on another scale. What would be possible is to use the observed effect size (a measure that quantifies the difference between groups) and calculate the sample size so that a difference of the observed magnitude is detectable for a certain power value. However, there is no well-established method for sizing the sample using the Kruskal-Wallis test, and what we did was to use the metric version of this comparison, which is via the variance analysis (ANOVA) model. After this calculation, the observed effect size is an η^2^ (eta squared) of 0.02, which is quite small. For a difference of this magnitude to be detectable in an ANOVA model with a type I error of 5% and a test power of 80%, it is necessary to observe at least 447 subjects in total (149 per group). We therefore have a limitation, as this is much more than the sample we have, in addition to suffering from the effect of very high dispersion.

Youden’s method determines that the cutoff point was found to maximize the sum of the class accuracies, and since we have three groups, we need two cutoff points to separate them, and here we show both (Table 4).

**Figure 5:**
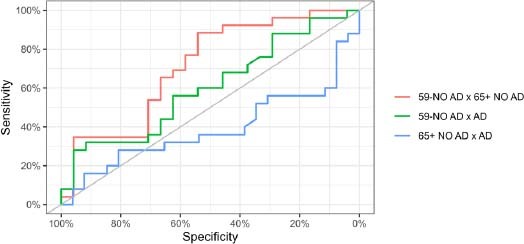
Receiver Operating Characteristic (ROC) Analysis.

Figure 5 shows the three Receiver Operating Characteristic (ROC) curves for each comparison, and we also present a search for cutoff points to maximize class accuracies, highlighting the generalization of sensitivity/specificity for more than two groups; we note that the comparison of the control group over 65 years of age with the AD control group is not satisfactory.

There is some very interesting evidence in our study, the fact that changes in pTau are much more expressive than those in t-tau, indicating that the best biomarker of tau is probably its phosphorylated form.

When evaluating the concentration of tau protein in the saliva of participants, a reduction in the expression of t-tau and an increase in salivary p-Tau were observed in patients with a probable diagnosis of Alzheimer’s dementia, demonstrating the viability of using these biomarkers^9^.

We encountered some obstacles in carrying out our study, which may be related to issues such as the time of day and/or metabolic or pathological factors, as we know that, for example, the elderly produce a smaller amount of saliva and this may interfere, perhaps, in the expression of some proteins. Huan et al. explain that the amounts of analyte in saliva are often lower than those in blood and that they can be influenced by a few factors, such as health conditions, stressful situations and diurnal/circadian oscillations^43^.

Laboratory methodologies to verify Aβ, tau, and Thr181P cerebrospinal fluid (CSF) levels, such as traditional singlex ELISA and luminex-based multiplex ELISA, are well established^37,44,45^. Compared to normal controls, our tests consistently show that p-Tau levels are higher in AD patients.

Salivary tau protein concentrations and AD pathophysiology show a direct relationship. Early evidence that tau levels are increased in individuals with AD may indicate an effective potential for diagnosing this irreversible dementia^38^. The expression of salivary p-Tau in patients with AD was significantly higher, almost 3.8 times more, than in individuals who do not have an Alzheimer’s diagnosis. This discovery significantly reinforces the hypothesis that p-Tau can be used as a biomarker for identifying Alzheimer’s disease.

Studies investigating pTau and t-tau proteins present in saliva followed 181 individuals with AD, 123 individuals with amnesia and MCI, 20 subjects with Parkinson’s disease, 16 individuals with FTD and 317 healthy controls. An elevated pTau/t-tau ratio was identified in patients with AD; in addition, one of the studies reported an increase in pTau / t-tau^38^ ratio using Western blot analysis for the phosphorylation sites S396, S404, T404, and a combination S400 and T403 (p<0.05) with increased median pTau / t-tau ratio at the S396 phosphorylation site (p<0.05). Although pTau and t-tau were described as increased in both studies, there was no reported statistical significance.

Our results are consistent with those of Shi et al., who reported an increase in pTau in AD patients compared to healthy individuals, reinforcing the hypothesis of the feasibility of salivary pTau analysis. These authors suggest that an increase in salivary pTau level is consistent with changes in the brain and CSF of patients with AD. This discovery indicates that the concentrations of t-tau protein and the phosphorylated forms can be antagonistic in saliva compared to other patient fluids, such as blood, cerebrospinal fluid, etc. The mean age of the study volunteers was within the expected range for AD, with 48 and 52% of the sample composed of individuals without and with AD, respectively. The mean age of the patients is within the age group in which the prevalence of AD starts to grow exponentially^3^.

Alternatives such as blood-based biomarkers have had a more significant impact on screening tools; in addition, panels of biomarkers perform better than single markers, such as proteins. This is due to sensitivity and specificity for diagnosis and prognosis^33,46,47^. The clinical diagnosis of Alzheimer’s dementia must be optimized without becoming expensive, that is, economically unviable. Furthermore, it needs to evolve so that it is conclusive and not performed with the aim of excluding possibilities^48^.

Several correlations have been proposed between the levels of t-tau and pTau in these fluids of the human body. Agnello et al. found a positive correlation between CSF and plasma pTau levels and CSF and plasma Aβ42/40 ratio levels. The analysis also indicated significant correlations, both positive and negative, between different analytes in the same matrix (e.g., CSF pTau vs. CSF Aβ42/40 ratio) or between different analytes in different matrices. As incredible as it may seem, no significant correlations were found between saliva and CSF or saliva and plasma, for all analytes considered^49^. These findings reinforce the idea that studies analyzing salivary proteins in AD can subsequently be used to establish patterns that will subsequently serve as correlations with blood and CSF.

It is currently estimated that two in three people living with dementia (PLWD) reside in low- and middle-income countries (LMIC), and the major concern is that the number of PLWD in these countries is expected to more than double in the coming years^50^. Our group believes that the high prevalence of AD in poor and middle-income countries justifies the urgent need to find inexpensive and minimally invasive diagnostic tests to detect biomarkers at early stages, where there are no symptoms of Alzheimer’s dementia. In this case, a saliva test, even if used as an initial screening method, could be extremely important, even as an element of some kind of public policy. Primary health care (PHC) could create initial assessment protocols, prioritizing patients with MCI and/or dementia using rapid tests, such as saliva tests, capable of identifying elderly people in the prodromal phase of AD.

In conclusion, our results indicate increased salivary expression of pTau in patients with probable AD, demonstrating the feasibility of using these molecules. The research participants were of varying ages, reinforcing that tau protein levels may be associated with different stages of the disease. We evaluated the sensitivity and specificity of pTAU as a biomarker using a ROC analysis, with satisfactory results for the younger groups, but not so good for the elderly, with and without AD. Therefore, we can also conclude that some variables need to be adjusted in the “elderly” groups in future investigations, among them other neurodegenerative conditions: cognitive assessments, memory tests, behavioral changes. Our methodology used an existing assay with some modifications, therefore requiring adaptations in the method, with the appropriate changes in the fluid, in this case, saliva. Using molecular biology, with more assertive resolution techniques, it is possible to detect salivary levels of tau protein capable of signaling preclinical conditions for AD.
